# Differences in Sentinel lymph node biopsy outcomes and prognosis between HER2-low and HER2-zero breast cancer

**DOI:** 10.1186/s12885-025-14970-8

**Published:** 2025-10-06

**Authors:** Koji Takada, Shinichiro Kashiwagi, Mariko Nishikawa, Asuka Kochi, Chika Watanabe, Haruhito Kinoshita, Kana Ogisawa, Masatsune Shibutani, Tamami Morisaki

**Affiliations:** 1 Department of Breast Surgical Oncology, Graduate School of Medicine, Osaka Metropolitan University, 1-4-3 Asahi-machi, Abeno-ku, Osaka, 545-8585 Japan; 2 Department of Gastrointestinal Surgery, Graduate School of Medicine, Osaka Metropolitan University, 1-4-3 Asahi-machi, Abeno-ku, Osaka, 545-8585 Japan

**Keywords:** Breast cancer, HER2-low, Prognosis, Sentinel lymph node, Axillary lymph node dissection

## Abstract

**Purpose:**

Human epidermal growth factor receptor 2 (HER2)-low breast cancer has been recognized as a distinct biological subset within HER2-negative breast cancer. This study aimed to examine the differences in sentinel lymph node metastasis (SLNM) rates and prognosis between HER2-low and HER2-zero breast cancers.

**Methods:**

This retrospective study evaluated 965 estrogen receptor-positive, HER2-negative breast cancer patients who underwent sentinel lymph node biopsy at Osaka Metropolitan University Hospital. Clinicopathological characteristics, SLNM rates, and prognostic outcomes were compared between patients with HER2-low and with HER2-zero breast cancers.

**Results:**

The SLNM rate was significantly higher in the HER2-low group than in the HER2-zero group (*p* = 0.039). However, disease-free survival (DFS), recurrence-free interval (RFI), overall survival, and breast cancer-specific survival were not significantly different between the two groups. In subgroup analysis excluding macrometastases, DFS and RFI were significantly longer in the HER2-low breast cancer group.

**Conclusion:**

HER2-low breast cancer exhibits a higher SLNM rate, suggesting unique biological behavior. However, its overall prognosis remains similar to that of HER2-zero breast cancer, with potential prognostic advantages in select subgroups.

## Background

Human epidermal growth factor receptor 2 (HER2) overexpression in breast cancer has historically been associated with poor prognosis. However, chemotherapy using anti-HER2 molecular targeted drugs, such as trastuzumab and pertuzumab, has achieved high therapeutic efficacy [1, 2]. Confirming breast cancer biology through immunohistochemistry (IHC) staining or in situ hybridization (ISH) is essential in selecting drug treatment strategies. New antibody-drug conjugates have demonstrated high therapeutic efficacy even in HER2-low breast cancers, which are not classified as HER2-positive, introducing a new concept of HER2 expression [3–5]. Breast cancers that are IHC 1 + or IHC 2 + without HER2 amplification on ISH were previously considered a subset of HER2-negative breast cancer; however, they are now classified as “HER2-low” breast cancer [3–5]. Conversely, HER2-negative breast cancers not classified as HER2-low are now classified as “HER2-zero.” Numerous reports have highlighted differences in prognosis, clinical characteristics, and biological features between these categories. For instance, compared with patients with HER2-zero breast cancer, those with HER2-low breast cancer tend to be older [4, 6–11] and exhibit higher estrogen receptor (ER) expression and lower Ki67 levels [4, 6, 8, 11–21]. Additionally, HER2-low breast cancer is associated with a higher incidence of lymph node metastasis [4, 7, 16, 22].

This study aimed to investigate differences in sentinel lymph node metastasis (SLNM) rates and prognosis between HER2-low and HER2-zero breast cancer. We hypothesized that the frequency of sentinel lymph node metastasis (SLNM) might differ between HER2-low and HER2-zero breast cancers.

## Methods

### Study design and ethics

This retrospective study was approved by the Ethics Committee of Osaka Metropolitan University (approve number: #926) and was conducted in accordance with the Declaration of Helsinki. All patients were informed of the investigational nature of this study and provided their written informed consent.

### Patients

A total of 965 patients diagnosed with ER-positive and HER2-negative breast cancer without distant metastasis or axillary lymph node metastasis were evaluated. All patients underwent sentinel lymph node biopsy (SLNB) during curative breast cancer surgery at Osaka Metropolitan University Hospital between April 2007 and December 2021. Diagnoses were made using specimens obtained via core needle biopsy or vacuum-assisted biopsy. Breast cancers meeting these criteria were designated as ER + HER2 BC. HER2 expression was assessed using the HercepTest II (Dako). HER2-zero tumors were defined as IHC 0 tumors, meaning no staining is observed or membrane staining that is incomplete and is faint/barely perceptible and in ≤ 10% of tumor cells, while HER2-low tumors were defined as those with other HER2-negative results [3–5]. If the diagnosis was IHC2, an additional evaluation using ISH was performed to confirm the negative result.

### Assessment and treatment

Staging evaluations included computed tomography, ultrasonography, and bone scintigraphy to confirm the absence of distant or axillary lymph node metastases. Surgical options included mastectomy or breast-conserving surgery, with SLNB performed using a dye-and-isotope technique [23, 24]. Sentinel lymph nodes were sliced into 2-mm-thick *serial* sections for pathological evaluation of metastases [25, 26]. Metastases were classified as macrometastases (> 2 mm), micrometastases (tumor diameter 0.2–2 mm or < 200 cells), or isolated tumor cells (< 0.2 mm or < 200 cells) [27]. Patients with macrometastases typically underwent additional axillary dissection.

Postoperative treatments were planned based on surgical procedures, disease subtypes, and disease stages. Disease-free survival (DFS) was defined as the interval between surgery and breast cancer recurrence or death from any cause. Recurrence-free interval (RFI) referred to the time between surgery and breast cancer recurrence. Overall survival (OS) and breast cancer-specific survival (BCSS) were defined as the intervals between surgery and death from any cause and from breast cancer, respectively. The patients were followed up for a median of 2349 days (range: 20–5959 days).

### Statistical analysis

Correlations between clinicopathological features were assessed using Pearson’s chi-square test. Odds ratio (OR) and 95% confidence intervals (CIs) were calculated by logistic analysis, and multivariable analysis was performed by the multivariable logistic regression model. Survival curves were generated using the Kaplan-Meier method and compared between groups using log-rank tests. Cox proportional hazards models were used to calculate hazard ratios (HRs) and 95% *CIs*, with multivariate analyses conducted via Cox regression. All statistical analyses were performed using SPSS version 28.0 (IBM Inc.). A p-value of < 0.05 was considered significant.

## Results

### Patient characteristics

The median patient age was 60 years (range: 30–91 years). The median tumor diameter was 15.1 mm (range: 3.2–74.4 mm). Progesterone receptor (PgR)-positive breast cancer was found in 589 patients (61.0%), and Ki67 levels were elevated in 188 patients (19.5%). Regarding HER2 expression, 555 patients (57.5%) were classified with HER2-low disease, while 410 patients (42.5%) were classified with HER2-zero disease. A total of 733 patients (76.0%) had a body mass index (BMI) of ≤ 25 kg/m^2^, while 56 patients (5.8%) had a BMI of ≥ 30 kg/m^2^. Additionally, 710 patients (73.6%) were never-smokers, whereas 90 patients (9.3%) were heavy smokers, with a smoking history of more than 20 pack-years. Table 1 presents the clinicopathological characteristics of the patients.

**Table 1:** Clinicopathological features of 965 ER + HER2-BC patients who underwent surgery combined with SLNB without preoperative chemotherapy

Parameters	Number of patients (*n* = 965) (%)
Age at operation (years old)	median 60 (range, 30–91)
Tumor size (mm)	median 15.1 (range, 3.2–74.4)
Progesterone receptor Negative/Positive	376 (39.0%)/589 (61.0%)
HER2 status HER2-zero/HER2-low	410 (42.5%)/555 (57.5%)
Ki67 ≤ 20%/>20%	777 (80.5%)/188 (19.5%)
Pathological axillary lymph node metastasis No metastasis/Only isolated tumor cell/Only micrometastasis/Metastasis	786 (81.4%)/6 (0.6%)/44 (4.6%)/129 (13.4%)
Lymph vascular invasion No/Yes	708 (73.4%)/257 (26.6%)
Nuclear grade 1/2/3	595 (61.7%)/285 (29.5%)/85 (8.8%)
Adjuvant chemotherapy No/Yes	865 (89.6%)/100 (10.4%)
Body mass index (kg/m^2^) ≤25/25 < and ≤ 30/<30	733 (76.0%)/176 (18.2%)/56 (5.8%)
Smoker (packs-years) 0/0 < and ≤ 20/20<	710 (73.6%)/165 (17.1%)/90 (9.3%)

*ER + HER2-BC* estrogen receptor-positive and human epidermal growth factor receptor 2-negative breast cancer, *SLNB* sentinel lymph node biopsy, *HER2* human epidermal growth factor receptor 2

Postoperative pathological findings revealed no axillary lymph node metastasis (ALNM) in 836 patients (86.6%), including 6 patients (0.6%) with isolated tumor cells and 44 patients (4.6%) with micrometastases. Lymphovascular invasion (LVI) was observed in 257 patients (26.6%). Nuclear grade (NG) assessment showed that 595 (61.7%) patients had Grade 1 disease, while 85 (8.8%) patients had Grade 3 disease. Postoperative chemotherapy was administered in 100 patients (10.4%).

### Correlation between ALNM and clinicopathological factors

Table 2 summarizes the correlation between ALNM and clinicopathological factors. A tumor diameter exceeding 20 mm was associated with a significantly higher frequency of metastasis (*p* < 0.001). Moreover, higher Ki67 values were associated with a significantly higher incidence of metastasis than were lower values (*p* = 0.005). Postoperative pathology revealed that SLNM was significantly more frequent in patients with LVI or high NG (LVI: *p* < 0.001, NG: *p* = 0.001). Although there was a trend toward increased metastasis in patients with a higher BMI, this was not significant (*p* = 0.077). Notably, metastasis was significantly more frequent in the HER2-low group than in HER2-zero group (*p* = 0.039).

**Table 2:** Correlation between axillary lymph node metastasis and clinicopathological features

Parameters	Axillary lymph node metastasis		*p* value
	No metastasis, including even micrometasis (*n* = 836)	Metastasis (*n* = 129)	
Age at operation (years old) ≤ 60 >60	435 (52.0%) 401 (48.0%)	65 (50.4%) 64 (49.6%)	0.728
Tumor size (mm) ≤ 20.0 >20.0	637 (76.2%) 199 (23.8%)	77 (59.7%) 52 (40.3%)	< 0.001
Progesterone receptor Negative Positive	330 (39.5%) 506 (60.5%)	46 (35.7%) 83 (64.3%)	0.408
HER2 status HER2-zero HER2-low	366 (43.8%) 470 (56.2%)	44 (34.1%) 85 (65.9%)	0.039
Ki67 ≤20% >20%	685 (81.9%) 151 (18.1%)	92 (71.3%) 37 (28.7%)	0.005
Lymph vascular invasion No Yes	649 (77.6%) 187 (22.4%)	59 (45.7%) 70 (54.3%)	< 0.001
Nuclear grade 1, 2 3	772 (92.3%) 64 (7.7%)	108 (83.7%) 21 (16.3%)	0.001
Adjuvant chemotherapy No Yes	780 (93.3%) 56 (6.7%)	85 (65.9%) 44 (34.1%)	< 0.001
Body mass index (kg/m^2^) ≤25 >25	643 (76.9%) 193 (23.1%)	90 (69.8%) 39 (30.2%)	0.077
Packs-years (packs-years) 0 >0	619 (74.0%) 217 (26.0%)	91 (70.5%) 38 (29.5%)	0.401

*HER2* human epidermal growth factor receptor 2

We examined the factors causing ALNM; tumor size (p < 0.001, OR = 2.162), Ki67 (p = 0.005, OR = 1.824), LVI (p < 0.001, OR = 4.118), NG (p = 0.002, OR = 2.345) and adjuvant chemotherapy (p < 0.001, OR = 7.210), as well as HER2-low (p = 0.040, OR = 1.504) were the factors associated with ALNM (Table 3). Even after performing the multivariate analysis, these remained independent factors (LVI: p < 0.001, OR = 2.892; adjuvant chemotherapy: p < 0.001, OR = 5.104; HER2-low: p = 0.042, OR = 1.556).

**Table 3:** Univariate and multivariate analysis with axillary lymph node metastasis

	Univarite analysis			Multivariate analysis		
Parameters	Odds ratio	95% CI	*p* value	Odds ratio	95% CI	*p* value
Age (years old) ≤ 60 vs. > 60	1.068	0.737–1.548	0.728			
Tumor size (mm) ≤ 20.0 vs. > 20.0	2.162	1.469–3.180	< 0.001	1.413	0.922–2.168	0.113
Progesterone receptor Negative vs. Positive	1.177	0.800-1.731	0.409			
HER2 status HER2-zero vs. HER2-low	1.504	1.020–2.219	0.040	1.556	1.015–2.384	0.042
Ki67 ≤20% vs. > 20%	1.824	1.198–2.777	0.005	1.043	0.637–1.707	0.867
Lymph vascular invasion No vs. Yes	4.118	2.808–6.037	< 0.001	2.892	1.887–4.434	< 0.001
Nuclear grade 1 or 2 vs. 3	2.345	1.377–3.995	0.002	0.698	0.355–1.371	0.297
Adjuvant chemotherapy No vs. Yes	7.210	4.579–11.352	< 0.001	5.104	2.929–8.894	< 0.001
Body mass index (kg/m2) ≤25 vs. > 25	1.444	0.959–2.173	0.078	1.463	0.938–2.282	0.093
Packs-years (packs-years) 0 vs. > 0	1.191	0.791–1.793	0.402			

*HER2* Human epidermal growth factor receptor 2, *HR* Hazard ratio, *CI* Confidence intervals

### Correlation between HER2 status and clinicopathological factors

The correlation between HER2 status and clinicopathological factors is shown in Table 4. HER2-low breast cancer was significantly associated with negative PgR expression (*p* = 0.012) and higher Ki67 levels (*p* < 0.001). Additionally, patients with a history of smoking were more likely to have HER2-low breast cancer (*p* = 0.068).

**Table 4:** Correlation between HER2 status and clinicopathological features

Parameters	HER2 status		*p* value
	HER2-zero (*n* = 410)	HER2-low (*n* = 555)	
Age at operation (years old) ≤ 60 >60	203 (49.5%) 207 (50.5%)	297 (53.5%) 258 (46.5%)	0.219
Tumor size (mm) ≤ 20.0 >20.0	313 (76.3%) 97 (23.7%)	401 (72.3%) 154 (27.7%)	0.152
Progesterone receptor Negative Positive	141 (34.4%) 269 (65.6%)	235 (42.3%) 320 (57.7%)	0.012
Ki67 ≤20% >20%	368 (89.8%) 42 (10.2%)	409 (73.7%) 146 (26.3%)	< 0.001
Lymph vascular invasion No Yes	292 (71.2%) 118 (28.8%)	416 (75.0%) 139 (25.0%)	0.194
Nuclear grade 1, 2 3	381 (92.9%) 29 (7.1%)	499 (89.9%) 56 (10.1%)	0.102
Adjuvant chemotherapy No Yes	372 (90.7%) 38 (9.3%)	493 (88.8%) 62 (11.2%)	0.338
Body mass index (kg/m^2^) ≤25 >25	308 (75.1%) 102 (24.9%)	425 (76.6%) 130 (23.4%)	0.601
Packs-years (packs-years) 0 >0	314 (76.6%) 96 (23.4%)	396 (71.4%) 159 (28.6%)	0.068

*HER2* Human epidermal growth factor receptor 2

### Prognostic differences according to HER2 status

**Fig. 1:**
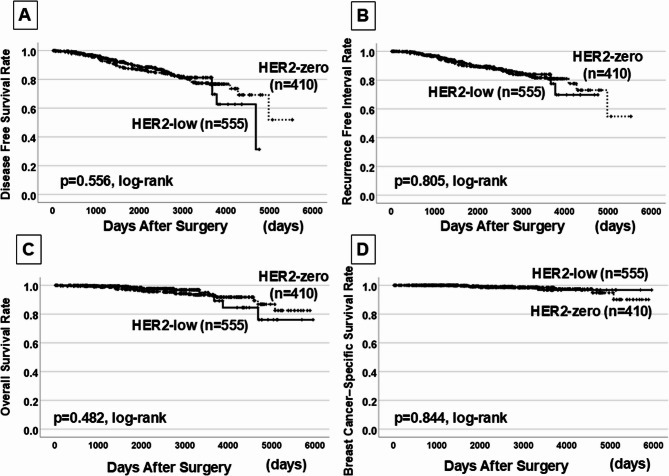
Kaplan-Meier curves for survival outcomes in HER2-low (*n* = 555) and HER2-zero (*n* = 410) breast cancer patients. **A** Disease-free survival (DFS), (**B**) Recurrence-free interval (RFI), (**C**) Overall survival (OS), (**D**) Breast cancer-specific survival (BCSS). No significant differences observed (log-rank test)

**Fig. 2:**
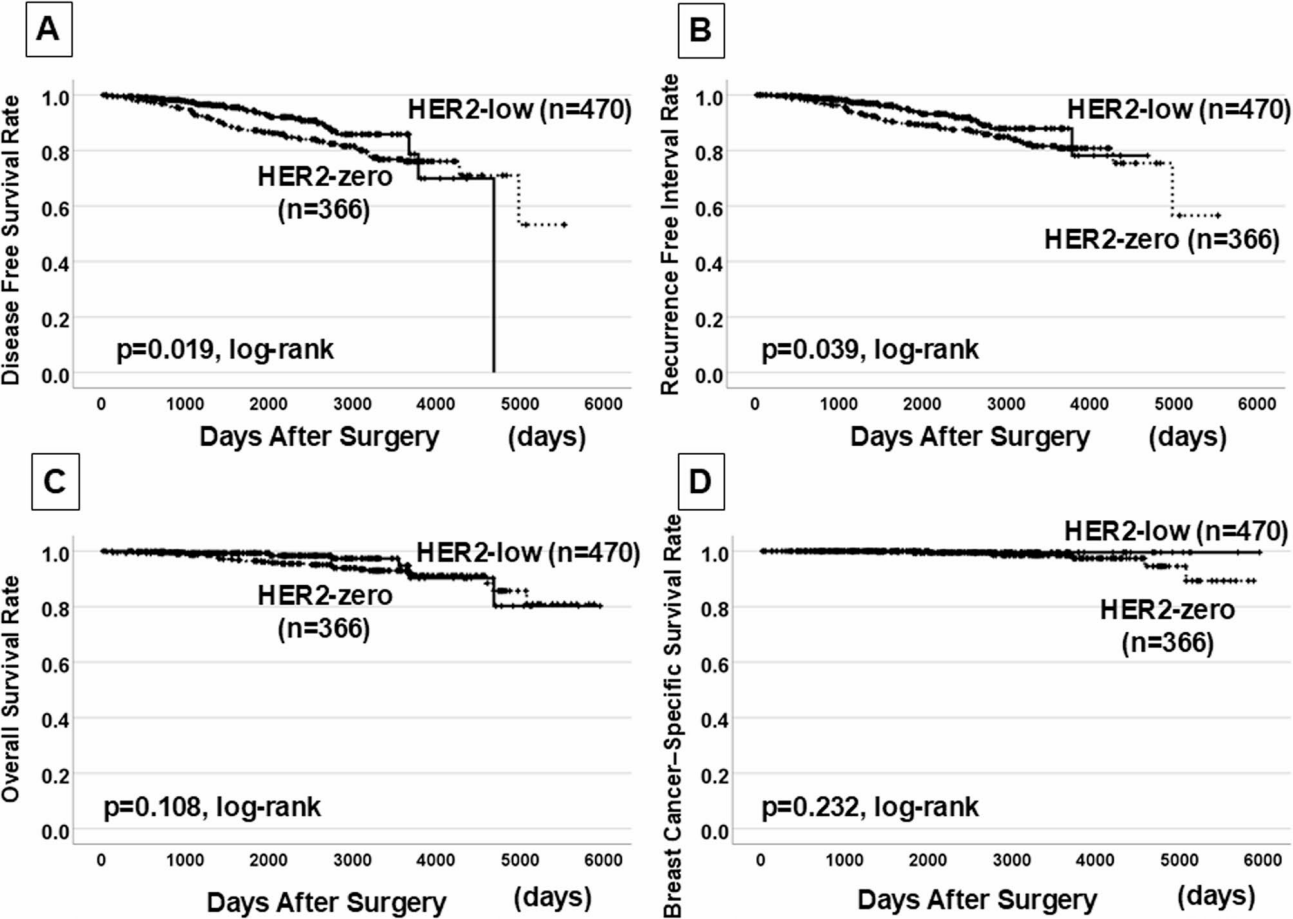
Survival analysis in patients without macrometastases (HER2-low: *n* = 470, HER2-zero: *n* = 366). DFS (A) and RFI (B) were significantly longer in HER2-low patients (*p* = 0.019, *p* = 0.039), while OS (C) and BCSS (D) showed no difference All figures are original and created by the authors

Log-rank tests showed that HER2 expression did not significantly affect DFS (*p* = 0.556), RFI (*p* = 0.805), OS (*p* = 0.482), and BCSS (*p* = 0.844) (Fig. 1). Similarly, univariate analysis revealed no significant differences in prognosis based on HER2 expression (DFS: *p* = 0.556, HR = 0.901; RFI: *p* = 0.176, HR = 1.383; OS: *p* = 0.483, HR = 0.787; BCSS: *p* = 0.844, HR = 1.123) (Table 5). However, in log-rank test analysis of the prognosis of 836 patients without macrometastases, OS and BCSS did not significantly differ based on HER2 expression (*p* = 0.108 and *p* = 0.232, respectively). Meanwhile, DFS and RFI were significantly longer in the HER2-low group than in the HER2-zero group (*p* = 0.019 and *p* = 0.039, respectively) (Fig. 2).

**Table 5:** Univariate and multivariate analysis with respect to each prognosis in 965 ER + HER2-BC patients who underwent surgery combined with SLNB without preoperative chemotherapy

	Disease-free survival						Recurrence-free interval					
	Univarite analysis			Multivariate analysis			Univarite analysis			Multivariate analysis		
Parameters	HRs	95% CI	*p* value	HRs	95% CI	*p* value	HRs	95% CI	*p* value	HRs	95% CI	*p* value
Age (years old) ≤ 60 vs. > 60	0.887	0.631–1.245	0.488				0.656	0.447–0.963	0.031	0.646	0.439–0.953	0.027
Tumor size (mm) ≤ 20.0 vs. > 20.0	2.425	1.723–3.411	< 0.001	2.060	1.451–2.925	< 0.001	2.861	1.973–4.147	< 0.001	2.471	1.685–3.622	< 0.001
Axillary lymph node metastasis No metastasis vs. Metastasis	2.081	1.393–3.109	< 0.001	1.679	1.074–2.624	0.023	2.558	1.683–3.887	< 0.001	2.121	1.334–3.372	0.001
Progesterone receptor Negative vs. Positive	1.199	0.838–1.714	0.321				1.265	0.851–1.881	0.244			
HER2 status HER2-zero vs. HER2-low	0.901	0.635–1.276	0.556				0.953	0.651–1.395	0.805			
Ki67 ≤20% vs. > 20%	1.366	0.887–2.102	0.156				1.383	0.865–2.213	0.176			
Lymph vascular invasion No vs. Yes	1.736	1.231–2.448	0.002	1.313	0.899–1.917	0.159	1.960	1.350–2.848	< 0.001	1.381	0.916–2.081	0.123
Nuclear grade 1 or 2 vs. 3	2.105	1.332–3.325	0.001	1.847	1.123–3.039	0.016	2.332	1.437–3.782	< 0.001	1.988	1.163–3.396	0.012
Adjuvant chemotherapy No vs. Yes	1.742	1.120–2.710	0.014	0.906	0.533–1.541	0.716	1.851	1.152–2.976	0.011	0.766	0.431–1.360	0.363
Body mass index (kg/m2) ≤25 vs. > 25	1.768	1.240–2.522	0.002	1.666	1.167–2.380	0.005	1.214	0.798–1.845	0.365			
Packs-years (packs-years) 0 vs. > 0	1.274	0.880–1.843	0.199				1.363	0.914–2.032	0.128			
	Overall survival						Breast cancer–specific survival					
	Univarite analysis			Multivariate analysis			Univarite analysis			Multivariate analysis		
Parameters	HRs	95% CI	*p* value	HRs	95% CI	*p* value	HRs	95% CI	*p* value	HRs	95% CI	*p* value
Age (years old) ≤ 60 vs. > 60	1.595	0.859–2.961	0.140				0.588	0.180–1.921	0.380			
Tumor size (mm) ≤ 20.0 vs. > 20.0	1.805	0.955–3.412	0.069	1.708	0.901–3.239	0.101	5.039	1.646–15.426	0.005	4.343	1.340-14.079	0.014
Axillary lymph node metastasis No metastasis vs. Metastasis	1.205	0.507–2.867	0.673				4.567	1.491–13.995	0.008	3.377	0.924–12.345	0.066
Progesterone receptor Negative vs. Positive	0.972	0.514–1.836	0.930				0.625	0.210–1.863	0.399			
HER2 status HER2-zero vs. HER2-low	0.787	0.403–1.538	0.483				1.123	0.353–3.574	0.844			
Ki67 ≤20% vs. > 20%	1.947	0.920–4.122	0.082	1.956	0.918–4.166	0.082	4.892	1.575–15.192	0.006	4.327	1.243–15.063	0.021
Lymph vascular invasion No vs. Yes	1.550	0.826–2.905	0.172				2.815	0.944–8.391	0.063	1.337	0.377–4.741	0.653
Nuclear grade 1 or 2 vs. 3	1.779	0.748–4.232	0.193				4.699	1.443–15.299	0.010	2.185	0.550–8.684	2.185
Adjuvant chemotherapy No vs. Yes	1.790	0.825–3.883	0.140				4.385	1.425–13.493	0.010	1.058	0.224–5.002	0.944
Body mass index (kg/m2) ≤25 vs. > 25	4.368	2.356–8.101	< 0.001	4.304	2.320–7.985	< 0.001	2.112	0.690–6.469	0.190			
Packs-years (packs-years) 0 vs. > 0	1.264	0.645–2.479	0.495				2.688	0.903–8.007	0.076	3.444	1.096–10.821	0.034

*ER + HER2-BC* Estrogen receptor-positive and human epidermal growth factor receptor 2-negative breast cancer, *SLNB* Sentinel lymph node biopsy, *HER2* Human epidermal growth factor receptor 2, *HR* Hazard ratio, *CI* Confidence intervals

Multivariate analysis of DFS showed that the independent prognostic factors were tumor size (*p* < 0.001, HR = 2.093), LVI (*p* = 0.035, HR = 1.548), BMI (*p* = 0.005, HR = 1.785), and HER2 expression (*p* = 0.040, HR = 0.646) (Table 6). Meanwhile, multivariate analysis of RFI identified age (*p* = 0.040, HR = 0.609), tumor size (*p* < 0.001, HR = 2.720), and HER2 expression (*p* = 0.043, HR = 0.616) as independent prognostic factors. HER2 expression was thus confirmed as an independent prognostic factor for RFI.

**Table 6:** Univariate and multivariate analysis with respect to each prognosis in 836 ER + HER2-BC with no axillary lymph node metastasis pathologically

	Disease-free survival						Recurrence-free interval					
	Univarite analysis			Multivariate analysis			Univarite analysis			Multivariate analysis		
Parameters	HRs	95% CI	*p* value	HRs	95% CI	*p* value	HRs	95% CI	*p* value	HRs	95% CI	*p* value
Age (years old) ≤ 60 vs. > 60	0.845	0.573–1.246	0.396				0.565	0.356–0.894	0.015	0.609	0.380–0.979	0.040
Tumor size (mm) ≤ 20.0 vs. > 20.0	2.363	1.593–3.504	< 0.001	2.093	1.396–3.136	< 0.001	2.811	1.816–4.351	< 0.001	2.720	1.744–4.242	< 0.001
Axillary lymph node metastasis No metastasis/ITC or micrometastasis	1.719	0.895–3.303	0.104				2.227	1.148–4.318	0.018	1.821	0.908–3.655	0.092
Progesterone receptor Negative vs. Positive	1.565	1.019–2.402	0.041	1.458	0.948–2.244	0.086	1.874	1.132-3.100	0.015	1.570	0.937–2.632	0.087
HER2 status HER2-zero vs. HER2-low	0.611	0.403–0.926	0.020	0.646	0.425–0.981	0.040	0.615	0.386–0.980	0.041	0.616	0.386–0.986	0.043
Ki67 ≤20% vs. > 20%	0.982	0.567–1.701	0.948				0.897	0.474–1.698	0.739			
Lymph vascular invasion No vs. Yes	1.725	1.157–2.571	0.007	1.548	1.031–2.325	0.035	2.000	1.287–3.108	0.002	1.538	0.967–2.445	0.069
Nuclear grade 1 or 2 vs. 3	1.452	0.795–2.652	0.225				1.699	0.900-3.207	0.102			
Adjuvant chemotherapy No vs. Yes	0.872	0.405–1.878	0.726				0.779	0.315–1.926	0.589			
Body mass index (kg/m2) ≤25 vs. > 25	1.940	1.297–2.902	0.001	1.785	1.189–2.681	0.005	1.212	0.737–1.992	0.449			
Packs-years (packs-years) 0 vs. > 0	1.286	0.845–1.957	0.240				1.475	0.932–2.336	0.097	1.337	0.840–2.128	0.221
	Overall survival						Breast cancer–specific survival					
	Univarite analysis			Multivariate analysis			Univarite analysis			Multivariate analysis		
Parameters	HRs	95% CI	*p* value	HRs	95% CI	*p* value	HRs	95% CI	*p* value	HRs	95% CI	*p* value
Age (years old) ≤ 60 vs. > 60	1.720	0.877–3.370	0.114				0.484	0.097–2.427	0.378			
Tumor size (mm) ≤ 20.0 vs. > 20.0	1.824	0.907–3.669	0.092	1.652	0.819–3.335	0.161	5.943	1.419–24.901	0.015	6.231	1.443–26.906	0.014
Axillary lymph node metastasis No metastasis/ITC or micrometastasis	1.507	0.460–4.931	0.498				5.762	1.118–29.711	0.036	3.378	0.606–18.832	0.165
Progesterone receptor Negative vs. Positive	1.085	0.539–2.183	0.820				0.900	0.214–3.774	0.885			
HER2 status HER2-zero vs. HER2-low	0.531	0.243–1.160	0.113				0.293	0.035–2.464	0.259			
Ki67 ≤20% vs. > 20%	1.410	0.581–3.419	0.447				2.724	0.543–13.659	0.223			
Lymph vascular invasion No vs. Yes	1.504	0.747–3.028	0.253				2.917	0.725–11.738	0.132			
Nuclear grade 1 or 2 vs. 3	0.686	0.164–2.865	0.606				1.737	0.211–14.279	0.607			
Adjuvant chemotherapy No vs. Yes	1.467	0.517–4.165	0.472				3.367	0.671–16.900	0.140			
Body mass index (kg/m2) ≤25 vs. > 25	4.358	2.238–8.486	< 0.001	4.225	2.166–8.243	< 0.001	1.188	0.239–5.904	0.833			
Packs-years (packs-years) 0 vs. > 0	1.185	0.569–2.468	0.650				4.990	1.192–20.893	0.028	5.130	1.154–22.797	0.032

*ER + HER2-BC* Estrogen receptor-positive and human epidermal growth factor receptor 2-negative breast cancer, *SLNB* Sentinel lymph node biopsy, *HER2* Human epidermal growth factor receptor 2, *HR* Hazard ratio, *CI* Confidence intervals

## Discussion

Numerous studies have investigated risk factors for metastasis in SLNB, with most identifying tumor size and LVI as key risk factors for SLNM [28–35]. Additionally, pathological malignancy has been reported to be associated with an increased risk of SLNM [28–33, 35]. In the current study, SLNM was more frequently observed in breast tumors with larger sizes, higher Ki67 levels, LVI, and higher NG, consistent with previous findings. Although one study examined differences in HER2 expression in relation to SLNM [30], it only assessed whether HER2 was positive or negative. To our best knowledge, this study is the first to report differences in SLNM rates between HER2-low and HER2-zero breast cancers.

Regarding the association between HER2 expression and clinicopathological factors, our results particularly those concerning Ki67 levels, differed from those previously reported [6, 14, 16, 18, 20]. This discrepancy may be attributed to the fact that our study was limited to ER-positive breast cancer. Some studies have suggested that when patients are stratified by hormone receptor status, those with HER2-low breast cancer exhibit different clinicopathological characteristics [7, 36]. Moreover, other reports indicate that differences in hormone receptor expression can alter the associations of age and tumor size with HER2 status [16, 37]. *One study has suggested crosstalk between hormone receptors and HER2* [37]. *Furthermore*,* this study demonstrated that in hormone receptor-positive breast cancer*,* Ki67 levels were higher in HER2-low cases than in HER2-zero cases* [37]. *Although HER2 expression is closely related to other biology of breast cancer cells themselves*,* multivariate analysis in this study revealed that the intensity of HER2 expression may have an independent effect on the prognosis of hormone receptor-positive breast cancer.*

Several meta-analyses have suggested that among patients with hormone receptor-positive breast cancer, those with HER2-low disease have better prognosis than those with HER2-zero disease [7, 18, 38]. This may be due to differences in PAM50 gene expression between HER2-zero and HER2-low breast cancer [4, 39, 40]. However, numerous studies have reported no significant prognostic differences between HER2-low and HER2-zero breast cancers [6, 8, 13, 19, 21, 36, 41–44]. The current study also found no prognostic differences in the overall cohort undergoing SLNB; however, significant differences emerged when the analysis was limited to patients without SLNM. We speculate that this may be related to the reported chemotherapy resistance of HER2-low breast cancer [4, 39]. The inclusion of SLNM patients in our study likely increased the proportion of patients receiving chemotherapy, potentially influencing the results. *The efficacy of treatment varies depending on the intensity of HER2 expression*,* but the differential response to endocrine therapy and chemotherapy complicates the investigation of its impact on prognosis.*

A key limitation of this study is the variability in IHC antibodies used to assess HER2 expression. Although a single HER2 antibody was used in this study, previous reports indicated that HER2 scoring can vary depending on the antibody used [45]. Furthermore, discrepancies in HER2 expression assessment can arise even among experienced pathologists [4, 46]. *HER2 expression is related to the biology of other breast cancer cells*,* but is particularly strongly related to hormone receptor expression. If hormone receptor-negative breast cancer were examined*,* the results may be significantly different from those of this study. Regarding prognosis*,* postoperative drug treatment will mainly consist of chemotherapy*,* so the results will not be uniform*. Despite the issue of intratumoral HER2 heterogeneity, our findings suggest that ER+/HER2 − breast cancer may exhibit both aggressive biological characteristics and a favorable prognosis.

## Conclusions

HER2-low breast cancer is associated with a higher SLNM rate than HER2-zero breast cancer, indicating distinct biological behavior. However, overall prognosis remains similar. Among patients without macrometastases, those with HER2-low breast cancer have longer DFS and RFI, suggesting potential prognostic benefits.

## Data Availability

The datasets generated and analyzed during the current study are available in the Osaka Metropolitan University repository [https://ocu-omu.repo.nii.ac.jp/?page=1&size=20&sort=controlnumber] and can be accessed upon reasonable request.

## Abbreviations

BCSS: Breast cancer-specific survival
BMI: Body mass index
CI: Confidence interval
DFS: Disease-Free survival
ER: Estrogen receptor
HER2: Human epidermal growth factor receptor 2
HR: Hazard ratio
IHC: Immunohistochemistry
ISH: In Situ Hybridization
LVI: Lymphovascular invasion
NG: Nuclear grade
OS: Overall survival
PgR: Progesterone receptor
RFI: Recurrence-Free interval
SLNB: Sentinel lymph node biopsy
SLNM: Sentinel lymph node metastasis
